# Long-term survival with complete remission after hepatic arterial infusion chemotherapy for liver metastasis from gastric cancer: a case report

**DOI:** 10.1186/s12957-015-0686-3

**Published:** 2015-09-04

**Authors:** Takahiro Toyokawa, Masaichi Ohira, Katsunobu Sakurai, Ryosuke Amano, Naoshi Kubo, Hiroaki Tanaka, Kazuya Muguruma, Kosei Hirakawa

**Affiliations:** Department of Surgical Oncology, Osaka City University Graduate School of Medicine, 1-4-3 Asahimachi, Abeno-ku, Osaka, 545-8585 Japan

**Keywords:** Gastric cancer, Hepatic arterial infusion chemotherapy, Liver metastasis

## Abstract

We report a case of long-term survival with complete remission after hepatic arterial infusion chemotherapy (HAIC) for liver metastasis from gastric cancer. A 62-year-old man underwent radical distal gastrectomy with D2 lymphadenectomy for an advanced gastric cancer. Solitary liver metastasis in the S2/3 segment was detected 26 months after initial surgery. The patient underwent HAIC with systemic chemotherapy. Serum CEA levels rapidly decreased, and CT scan showed disappearance of the tumor with complete clinical response 8 months after HAIC. HAIC was performed 83 times in total, until the hepatic artery proper was adequately obstructed. No severe adverse effects were observed during HAIC treatment. The patient is still disease-free without further chemotherapy more than 12 years after HAIC. Our experience suggests that HAIC should be considered as a treatment option in patients with resectable liver metastasis from gastric cancer. However, further studies are needed to verify the validity of HAIC for resectable liver metastasis from gastric cancer.

## Background

Gastric cancer is the fourth most common malignancy worldwide [1]. Although the prognosis of gastric cancer has significantly improved because of improvements in disease diagnosis and treatment, the prognosis of recurrent gastric cancer remains poor [2]. Chemotherapy is regarded as the standard treatment for recurrent gastric cancer, although the median survival time of recurrent/metastatic gastric cancer treated by chemotherapy is reportedly only 12.5–13.8 months [3–6], which seems to be unsatisfactory. The liver is one of the most common sites of recurrence of gastric cancer; however, the treatment strategy for liver metastasis of gastric cancer has not yet been established. Here, we report a case of complete remission for more than 12 years following hepatic arterial infusion chemotherapy (HAIC) in a patient with solitary metachronous liver metastasis from gastric cancer. To the best of our knowledge, the present case is the longest survivor of liver metastasis from gastric cancer who was treated with HAIC without surgery.

## Case presentation

**Fig. 1:**
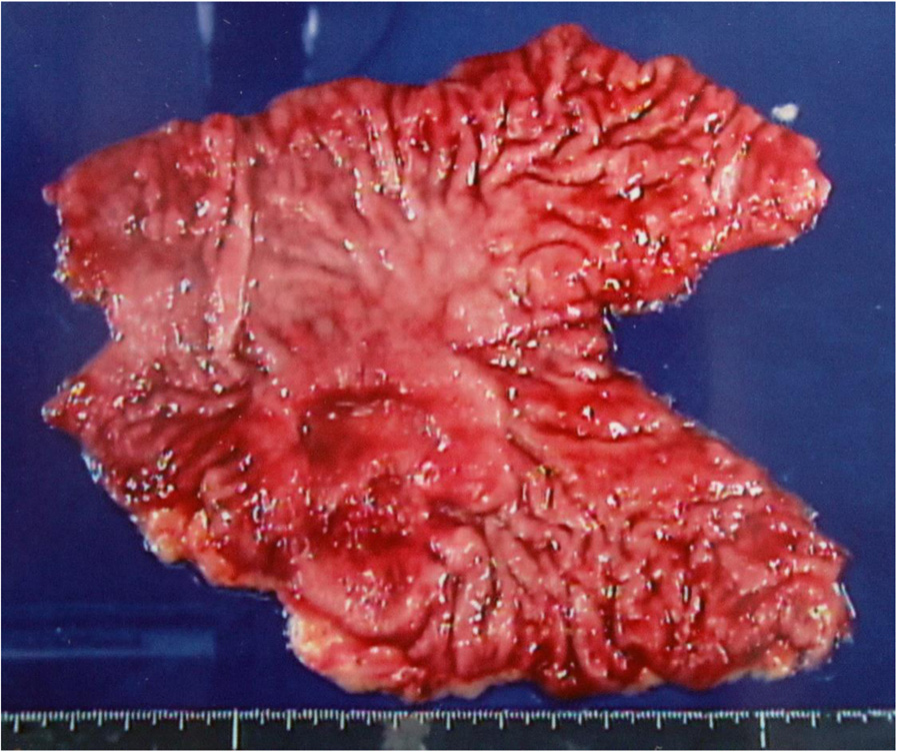
The resected specimen showing a type 3 tumor around the gastric antrum measuring approximately 55 × 50 mm in size

A 62-year-old man was referred to our institution in February 2000, after being diagnosed with gastric cancer as a result of medical examination for a 1-month history of heart burn at another hospital. His family and clinical histories were unremarkable. Laboratory data were within normal limits. Serum carcinoembryonic antigen (CEA) level was elevated to 185 ng/ml, but carbohydrate antigen (CA) 19–9 was 33 U/ml, which was within the normal range. Upper gastrointestinal endoscopy revealed a semi-circumferential type 3 tumor primarily located at the posterior wall of the gastric antrum. Histopathological examination in our institution confirmed moderately differentiated adenocarcinoma. Abdominal ultrasonography and abdominal computed tomography (CT) revealed no suspicious metastatic lesions in the liver, peritoneum, or lymph nodes. Intraoperatively, we did not find tumor invasion to adjacent organs, peritoneal dissemination, or liver metastasis. On the basis of these findings, the patient underwent radical distal gastrectomy with D2 lymphadenectomy and Billroth-I reconstruction in March 2000. The resected specimen showed a type 3 tumor that measured approximately 55 × 50 mm in size around the gastric antrum (Fig. 1). Microscopic examination revealed a moderately differentiated adenocarcinoma infiltrating the serosa, and the margin was negative for cancer. The final pathological diagnosis of the gastric cancer was T4aN3M0, Stage IIIC according to the third English edition of the Japanese Classification of Gastric Carcinoma [7]. His postoperative course was uneventful, and the patient was discharged on the 20th postoperative day. The serum CEA level decreased to a normal range within 3 months after surgery.

**Fig. 2:**
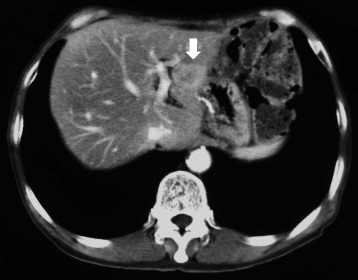
Abdominal computed tomography revealing a 15 mm metastatic lesion in the S2/3 segment of the liver (arrow)

**Fig. 3:**
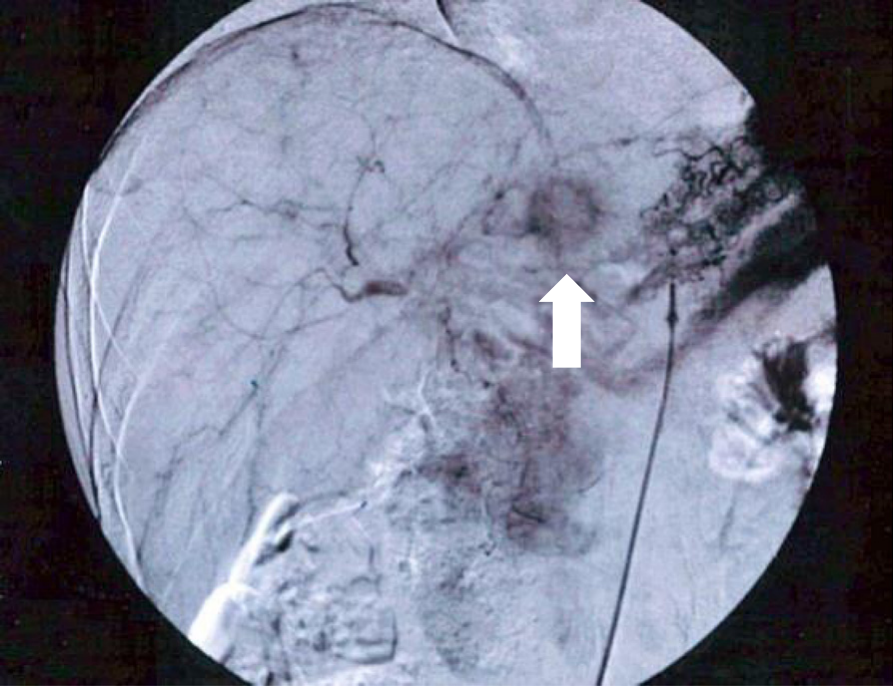
Angiography revealing the tumor stain in the lateral segment of the left liver lobe (arrow), consistent with CT scan findings

**Fig. 4:**
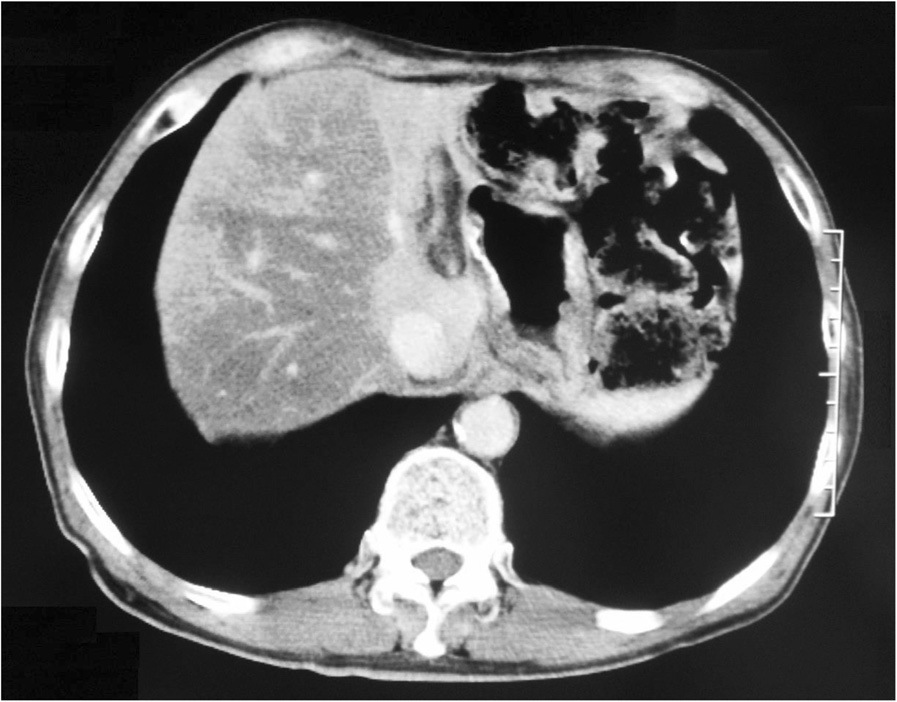
Abdominal computed tomography reveals that the tumor disappeared with atrophic change of the left lobe, together with a clinical complete response, 8 months after hepatic arterial infusion chemotherapy

**Fig. 5:**
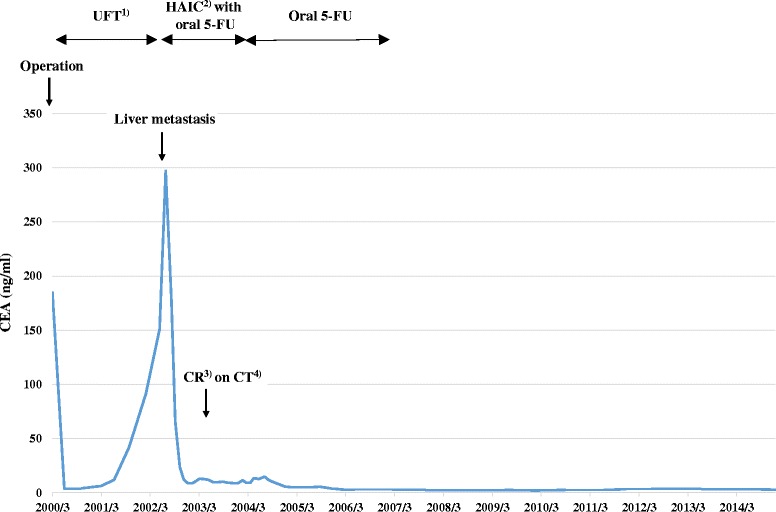
Clinical course and treatment of the patient. (1) Uracil-tegafur; (2) Hepatic arterial infusion chemotherapy; (3) Complete response; (4) Computed tomography

Following the operation, the patient started postoperative adjuvant chemotherapy with uracil-tegafur (300 mg/day; 100 mg three times daily after meals) from April 2000. However, about 15 months after the initial surgery, CEA levels began to gradually increase. The anticancer drug was changed to S-1, but he could only receive S-1 for 2 weeks due to appetite loss. CT scan performed in May 2002 revealed a 15-mm-sized metastatic lesion in the S2/3 segment of the liver, without any other suspicious lesions in the lymph nodes or peritoneum (Fig. 2). Angiography revealed staining suggestive of a tumor in the lateral segment of the left lobe of the liver, consistent with the results of the CT scan (Fig. 3). Diagnosing solitary liver metastasis, systemic chemotherapy using oral 5-FU (150 mg/day; 50 mg three times daily after meals) with HAIC (500 mg 5-FU for 2 h, weekly) from the proper hepatic artery through the femoral artery via a subcutaneously implanted port, was started in July 2002. Just before starting HAIC, CEA levels had increased to 297 ng/ml. After HAIC, CEA levels decreased rapidly and CT scan showed disappearance of the tumor with atrophic change of the left lobe, with a complete clinical response being obtained in March 2003 (8 months after commencement of HAIC) (Fig. 4). In total, HAIC was performed 83 times until April 2004, by which time the hepatic artery proper was obstructed. During HAIC treatment, no severe adverse effects were observed. After the catheter was removed, CEA levels reached the normal range in December 2004 (29 months after commencement of HAIC), and oral administration of 5-FU was completed in February 2007. Since completion of all chemotherapy, the patient has been following up periodically on an outpatient basis and is still disease-free with no further chemotherapy as of April 2015 (Fig. 5).

## Conclusions

The liver is one of the common sites of recurrence after curative surgery for gastric cancer. Among initial recurrence sites after curative gastrectomy, the liver was reported to account for 15.8–33.1 % of cases [8–10]. Yoo et al. [8] reported that in their study, 75 of 96 patients (78.1 %) with recurrence in the liver had no extrahepatic metastases. Liver metastases is considered a systemic disease that is not an indication for surgery with a curative intent, as the Japanese Gastric Cancer Treatment Guidelines state that chemotherapy is the treatment to be primarily considered for recurrent gastric cancer [11]. It is generally believed that chemotherapy cannot ultimately lead to complete cure even if patients are considered to have a complete response, and hence, the goal of chemotherapy is to prolong survival. In fact, although long-term survival after surgical resection for liver metastasis has been reported, few reports have documented long-term survival after HAIC for liver metastasis. To the best of our knowledge, this is the first report of a patient with long-term survival who experienced complete remission for more than 12 years following HAIC for liver metastasis from gastric cancer.

Since reports of the efficacy of systemic chemotherapies for gastric cancer [3, 4], the number of patients undergoing HAIC has been decreasing. HAIC is one of the effective local treatments for liver metastases and is considered for patients who have no extrahepatic metastasis and when the occurrence of hepatic metastasis is expected to determine the patient’s prognosis. Unlike systemic chemotherapy, HAIC can provide high concentrations of anticancer agents directly to the liver, resulting in a decrease in adverse drug effects. The reported response rates of HAIC are 71–83 % [12–14], which seems to be higher than those with systemic chemotherapy that have been reported in randomized controlled trials [2]. However, there is currently no established regimen of HAIC. Ojima et al. [14] reported that low dose 5-FU (500 mg, weekly), which was also used in the present case, could be used for a long period (the average treatment period of HAIC was 14.7 months) without severe adverse effects in 18 patients. Similarly, our patient underwent HAIC for 21 months without suffering any adverse effects. Since one of the disadvantages of HAIC is its inability to control extrahepatic tumor growth, we initially treated the patient with systemic chemotherapy. Although the standard regimen of systemic chemotherapy for metastatic gastric cancer in Japan was not yet established at the time, we decided to use oral 5-FU because the patient had not been able to tolerate S-1 due to a loss of appetite.

While the efficacy of curative resection for liver metastasis of colorectal cancer has been established [15], the significance of liver resection for metastasis from gastric cancer is still controversial. Although liver resection for metastases from gastric cancer is expected to be a potentially curative treatment, the results of some studies have been disappointing, suggesting that this type of surgery should be limited to certain patient subtypes as a part of multimodal treatment [16, 17]. Okano et al. [18] reported that solitary and metachronous metastases were independent favorable prognostic factors in patients who underwent hepatectomy for liver metastasis from gastric cancer. Others have also identified the same factors as independent favorable prognostic factors of liver resection for metastasis from gastric cancer [19–22]. Therefore, patients with these factors could be considered as potentially good candidates for curative liver resection. From this perspective, curative liver resection might have been indicated in our patient; however, because CT scan showed complete remission at the second follow-up examination after starting HAIC, together with rapidly decreasing CEA levels, we decided to continue HAIC, with which the complete remission persisted for more than 10 years even after the HAIC was interrupted. Similarly, Ojima et al. [14] reported one case that survived for more than 5 years without any signs of recurrence after treatment with HAIC. Although extremely rare, our case indicates that HAIC can lead to complete cure in selected patients with liver metastasis from gastric cancer.

Recurrence in the remnant liver after liver resection is observed most frequently within 2 years of the initial therapy, and no report has demonstrated the benefit of re-resection of the liver. These findings suggest that occult intrahepatic metastases may already exist at the time of the liver resection in most cases. Lambert et al. [23] advocated “a test of time” as an approach that identifies patients who are not likely to be cured by hepatic resection for liver metastases from colorectal cancer. This approach makes it possible to avoid unnecessary liver resections by reevaluating the development of intrahepatic or extrahepatic metastases 3–6 months later. In our case, HAIC with systemic chemotherapy prior to surgery led to complete remission, resulting in avoidance of liver resection. Hence, the “test of time” approach for resectable liver metastasis from gastric cancer may be a reasonable strategy.

We described long-term survival with complete remission in a patient who underwent HAIC for liver metastasis from gastric cancer. Our experience suggests that HAIC should be considered as a treatment option in patients with resectable liver metastasis from gastric cancer. However, further studies are needed to clarify which treatment, surgery, systemic chemotherapy, or HAIC is the most beneficial for resectable liver metastasis from gastric cancer.

## Consent

Written informed consent was obtained from the patient for publication of this case report and any accompanying images. A copy of the written consent is available for review by the Editor of this journal.
